# High Prevalence of Active Tuberculosis in Adults and Children with Idiopathic Inflammatory Myositis as Compared with Systemic Lupus Erythematosus in a Tuberculosis Endemic Country: Retrospective Data Review from a Tertiary Care Centre in India

**DOI:** 10.31138/mjr.32.2.134

**Published:** 2021-06-30

**Authors:** Latika Gupta, Rohit Aggarwal, R Naveen, Able Lawrence, Abhishek Zanwar, Durga Prasanna Misra, Vikas Agarwal, Ramnath Misra, Amita Aggarwal

**Affiliations:** 1 Department of Clinical Immunology & Rheumatology, Sanjay Gandhi Postgraduate Institute of Medical Sciences, Lucknow, India; 2 UPMC Myositis Centre, Division of Rheumatology and Clinical Immunology, University of Pittsburgh, Pittsburgh, PA, United States of America

**Keywords:** Myositis, lupus, tuberculosis, India, infections, dermatomyositis, glucocorticoids

## Abstract

**Aim:**

Infections are the leading cause of morbidity and mortality in idiopathic inflammatory myositis (IIM) with India being endemic for Tuberculosis (TB). We compared and contrasted the prevalence, clinical profile and outcomes of active TB in IIM with systemic lupus erythematosus (SLE).

**Methods:**

Medical records were reviewed for adults and children with IIM (Bohan and Peter criteria) and SLE (ACR criteria) at a tertiary care hospital in India from January 2015 to October 2017. Follow-up was recorded until February 2020 for all those who had developed active TB.

**Results:**

Of 167 (132 adults and 35 juvenile) IIM and 280 (131 adults and 149 juvenile) SLE, active TB occurred in 24 (14.4%) IIM (22 (16.7% of 132) adults; 2 (5.71% of 35) juvenile) and 18 (6.4%) SLE [(8 (6.1% of 131) adults; 10 (6.7% of 149) juvenile, p-value < 0.01]. Patients with IIM had higher odds of developing TB as compared with SLE [OR 2.24 (CI 1.5–5.5), p=0.007]. The risk of developing active TB was 68-fold and 30.4-fold higher in patients with IIM and SLE, respectively, as compared with the general population. Extrapulmonary forms were more common (14/24). Nearly half developed TB during active IIM, at a glucocorticoid dose of 0.25 (0–1.5) mg/kg/day. Over a follow-up duration of 27 months (8–184), all were cured of TB, though prolonged course of anti-tuberculous treatment was required in 25%, and five IIM relapsed during treatment.

**Conclusion:**

Patients with IIM have increased risk of active TB, with common extrapulmonary forms, slow response, and relapses during treatment.

## INTRODUCTION

A diagnosis of Idiopathic Inflammatory Myopathies (IIM) entails significant morbidity, and, at times, even mortality.^1^ In the developing world, infections are the leading contributor to such morbidity. Tuberculosis (TB) is one such infection, that remains a particular challenge in this part of the world. The emergence of drug-resistant strains has only added to the problem. Polypharmacy, in the setting of chronic illnesses, further compounds the problem.

Recent estimates suggest the prevalence of TB in India to be 3.2 cases per thousand population.^2^ Patients with IIM often require high doses of glucocorticoids for long periods. Poor cough reflex from pharyngeal and respiratory muscle involvement and immobility results in poor handling of both aerosols and oral secretions. Moreover, some patients IIM also have underlying complement pathway defects, such as Mannose Binding Lectin deficiency which is reasonably common and confers a higher risk of TB.^3,4^ Children are more likely to harbour such inherited deficiencies.^5^

Over the last two decades, public health initiatives in India have attempted to address the issues of rising prevalence, treatment default, and the emergence of drug resistance in TB with partial success.^2^ On the same lines, there have been efforts towards decreasing the usage of glucocorticoids in rheumatic disorders too. The changing dynamics of therapeutic practices could have a bearing on the prevalence of tuberculosis in these diseases, and also influence the ways this problem is addressed. Since a diagnosis of IIM entail long term treatment with high doses of glucocorticoids, we sought to determine the prevalence of active TB in adults and children with IIM and compare it with patients diagnosed with Systemic Lupus Erythematosus (SLE), another rheumatic disease that requires high dose glucocorticoids and are associated with higher incidence and prevalence of TB.^6,7^

## METHODS

Medical records from paper charts and electronic medical records were reviewed for adults and juvenile patients with definite and probable IIM by Bohan and Peter criteria 1975)^8^ following up at a tertiary care hospital in India from January 2015 to February 2020. The approach was all-inclusive irrespective of a past history of TB. Radiographs of the chest are usually obtained in all patients before beginning immunosuppression, ruling out the possibility of active pulmonary TB at initiation of immunosuppressant (IS) therapy. Patients with drug-induced myopathies, inherited or degenerative myopathies and endocrine, infectious, or metabolic muscle disorders were excluded. Institute ethical clearance for waiver of consent for retrospective data review was obtained [2017-41-IP-76] as per local guidelines.^9^ Demographic variables and clinical profiles, as well as disease activity, were noted. Records of adults and children with SLE (ACR criteria 1997) collected for a previous study were drawn for comparison with IIM.^10^

Assuming a confidence interval of 95% and a margin of error 2% with a 1.8% prevalence of TB in IIM from an-other study,^11^ the sample size was calculated to be 170. An event of TB was defined by either the demonstration of acid-fast bacilli (AFB) in the tissue or fluid obtained or presumptive diagnosis^12^ based on clinical and radiological features and improvement with anti-tuberculous therapy (ATT). Information on the organs involved, past or family history of TB, duration of ATT, drug interactions and adverse events, glucocorticoid and other immunosuppressive drugs received were obtained. A glucocorticoid (GC) dose >0.5 mg/kg was defined as high dose steroids. Details on outcomes such as cure of TB, resistance, relapses of TB and the primary disease, and death were recorded.

Active disease and relapse of IIM were defined as per physician assessment-based on new/subacute muscle weakness alongside elevated muscle enzymes or weakness requiring an increase in immunosuppression as assessed by the treating physician. A worsening of skin rash was also recorded as flare of disease in those with dermatomyositis or overlap myositis. The presence of interstitial lung disease (ILD) was defined as the presence of lung fibrosis by high-resolution computed tomography and evidence of restrictive physiology on pulmonary function tests. Screening for ILD was done only in those with breathlessness on exertion or crackles on respiratory examination. Chronic liver disease defined as a progressive deterioration of liver functions for more than six months, which includes synthetic functions, detoxification and excretory functions, assessed using a combination of ultrasound liver, liver function tests, coagulation profile and elastography.^13^ Overlap myositis defined by presence of myositis and fulfilling one CTD criteria.^14–18^ Antisynthetase syndrome defined by Connor’s criteria.^19^ Complete response, partial response and relapse in SLE as defined by EULAR/ERA-EDTA criteria.^20^

## STATISTICAL ANALYSIS

Comparisons were drawn between SLE and IIM patients. Descriptive statistics and non-parametric tests were used for comparisons. Results are expressed as median ± 2SD. The analysis was done using GraphPad Prism for Mac (trial version 7).

## RESULTS

The IIM cohort had 132 adults and 35 juvenile IIM patients with mean age at diagnosis 32 and eight years respectively (**Table 1**). The SLE cohort had 131 adult and 149 juvenile patients with mean age at diagnosis 32.4 and 13.7 years. Among adult IIM patients 42.4% (56) were DM, 21.2 % (28) PM, 9.8% (13) anti-synthetase, and 26.5% (35) overlap myositis. Among juvenile IIM patients, most were JDM 74.3% (26) (**Table 1**).

**Table 1.** Population demographics of SLE and IIM cohorts.

**Variabl**e	**Adult SLE**	**Pediatric SLE**	**Adult IIM**	**Juvenile IIM**
Patient number	131	149	132^*^	35^#^
Median follow up at the time of inclusion into the study (years)	3.0	6.0	5.5	8.0
Female: Male	8:1	9:1	6.3:1	1.5:1
Median age (years)	32.4	13.7	32	8
Lupus nephritis (%)	51.9	100	NA	NA
Chronic kidney disease (%)	4.6	7.3	0	0
Number of TB cases	8	10	22	2
Incidence rate/100 patient years follow up	2.0	1.1	2.3	1.9

* 56 DM, 28 PM, 13 Anti-synthetase syndrome and 35 Overlap myositis;

# 26 jDM, 1 PM and 8 Overlap Myositis

### The prevalence of tuberculosis in adults and children with IIM

Active TB was seen in 24 (14.4%) IIM patients (**Table 2**) with 22 (16.7%) being in adults and two (5.71%) in juvenile IIM cases. Of those with TB, 18 had DM; three had PM, four anti-synthetase syndrome and seven had overlap myositis.

**Table 2.** Clinical profile of patients with Tuberculosis.

**Clinical parameter**	**Adult SLE (n=8%)**	**Pediatric SLE (n=10%)**	**IIM (n=24%)**	**P (Adult SLE vs. IIM)**	**P (Pediatric SLE vs IIM)**

Cases on high dose steroid^#^	3 (37.5)	4 (40.0)	5 (20.8)	0.378	0.395

*Number of patients on immunosuppressive drugs*					
Prednisolone	7 (87.5)	10 (100)	22 (91.6)	1.00	1.00
MMF	1 (12.5)	2 (20)	2 (8.33)	1.00	0.5636
Cyclophosphamide	2 (25)	5 (50)	2 (8.33)	0.254	0.013
AZA	1 (12.5)	2 (20)	-	-	-
Methotrexate	1 (12.5)	-	5 (20.8)	1.00	-

Disease duration <1 year	4 (50)	4 (40)	8 (33.3)	0.432	0.713
Pulmonary: Extra-pulmonary	4:4	5:5	5:7	1.00	1.00
Tissue/Fluid diagnosis	1 (12.5)	7 (70)	10 (41.7)	0.209	0.258
Active Underlying disease	4 (50)	7 (70)	12 (50)	1.0	0.451

Relapse of TB	2^*^ (25)	0	2 (11.8)	0.254	1.00

Resistant Tuberculosis	0	0	2	1.00	1.00
MDR suspect Pre-XDR	0	0	1	1.00	1.00
Death	1 (12.5)	1 (12.5)	0	0.250	0.294

# (>0.5mg/kg)

* One patient each had CKD and family history of TB.

SLE: systemic lupus erythematosus; IIM: idiopathic inflammatory myopathies; MDR: multidrug resistant; XDR: extreme drug resistance; TB: tuberculosis.

P calculated using Fisher’s exact between groups.

**Figure 1.**
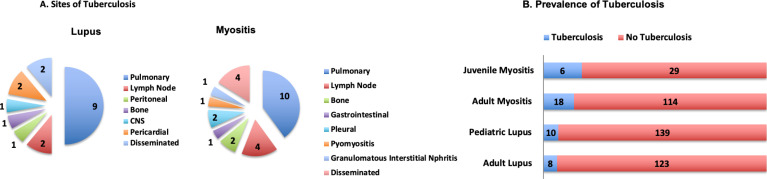
Sites of tuberculosis in SLE and IIM 1b. Prevalence of tuberculosis in SLE and IIM.

Lung was most common focus of TB, 10 patients had pulmonary TB and 2 patients had isolated pleural effusion. Extra-pulmonary TB was seen in 12 patients: Lymph node and disseminated TB was found in four patients each. Four patients had unusual site of involvement. Two had bone TB; one of them had pyomyositis in addition. One patient each had renal and gastrointestinal involvement (**Figure 1**).

TB occurred at median 2 (1–2.5) years after the onset of IIM in adults, and recurrence was seen in one of the 22 (4.5%) cases 5.5 years after complete treatment. On the other hand, TB was diagnosed at the disease onset in one of the two cases of jDM, with a recurrence of MDR sputum positive pulmonary TB 4.5 years later.

### Prevalence of tuberculosis in adults and children with SLE

One-hundred thirty-one adults and 149 children with SLE were included for comparison. Of the 131 (8:1 female:male) adult SLE patients (median age 32.4 years, mean follow-up 3 years, 68 (51.9) had lupus nephritis and 4.6 had chronic kidney disease) eight developed Tuberculosis (6.10%).

Half the patients developed TB within 2 years and a half were extra-pulmonary (1 lymph node, 1 peritoneal, 1 bone, 1 CNS). Of the 149 (female:male 9:1) paediatric SLE patients (median age 13.7 years, mean follow up 6 years, all had lupus nephritis) and 10 (6.71%) developed TB. Six of 10 had TB within six months of SLE diagnosis and half were extra-pulmonary (2 pericardial, 1 lymph node and 2 disseminated). One of them died of disseminated TB (**Figure 1**).

### Comparison of SLE and IIM patients with TB

The overall incidence of TB in IIM was 14.37/100 patients (2/100 patient years) and in SLE was 6.42/100 patients (2.386/100 patient years). The incidence of TB in adult IIM patient was 16.6/100 patients while in juvenile IIM was 5.71/100 patients. In SLE patients, incidence of TB is 6.1/100 patients in adults and 6.7/100 patients in children. Considering an annual TB rate of 211 per 100,000 of the general population,^2^ the risk of developing active TB was 68-fold higher in patients with IIM and 30.4-fold higher in those with SLE. Patients with IIM had higher odds of developing TB as compared with SLE [odds ratio 2.24 (CI 1.5–5.47), p=0.007).

The was no difference in the age, duration from disease onset to development of tuberculosis, the prevalence of extra-pulmonary TB or death due to TB between IIM and SLE (**Table 2**). Family history of TB was present in two children and four adults with SLE.

### Factors predicting TB

The IIM patients who developed TB were no different from those without TB with regard to the gender, type of IIM, or the age of onset of IIM (childhood versus adulthood). Most patients with IIM who developed TB [n=16 (66.7%)] developed various other infections as well (Table 2). Three of them had opportunistic infections, one each with cytomegalovirus, esophageal candidiasis, and herpes zoster. Eight patients (33.3%) had other risk factors for infections (ILD, Chronic Liver Disease, and pregnancy).

### Diagnosis of TB in IIM

In almost half the cases (n=13), the diagnosis was based on definite evidence of mycobacterial infection, i.e. smear evidence of acid-fast bacilli (n=7), histology (n=4), or growth in cultures (n=3) (**Table 3**). An empiric diagnosis was made in 11 patients. One patient was suspected to have miliary TB, but died before the diagnosis could be confirmed. One patient of DM had culture-proven pre-extremely drug-resistant (Pre-XDR) infection. She improved on therapy but suffered kanamycin induced hearing loss. IIM patients who developed TB had a median disease duration of 2 (0.2–12) years.

**Table 3.** Details of TB patients in IIM.

**Case number**	**Age, gender**	**IIM type + clinical features**	**Disease duration at the time of TB (years)**	**Site of TB**	**GC dose (mg/kg)**	**IS at the time of disease**	**Method of TB diagnosis**	**Duration of ATT (months)**	**Total follow up duration (months)**	**ADRs or Drug interactions**	**Other infections**	**Other**	**TB free interval after ATT (months)**	**Flare induced by ATT Y/N**	**ANA**	**ENA**
**Adult myositis**																
1 Su	21, F	DM Arthritis Myositis Alopecia Myocarditis Pneumomediastinum	0.25	P	1.0	None	Sputum smear, BACTEC (+)^”^	24, followed by prophylaxis	30	PAS pruritis, Amikacin induced hearing loss	Hospital acquired pneumonia	Pre-extremely drug resistant TB	6	Yes by TB not by ATT	Negative	Negative
2 Gu	22, F	OM (MCTD) Arthritis Myositis RP Rash	1.5	P	0.5	MTX	Empiric (CXR)	6	57	No		Bilateral shoulder dislocation	51	Yes	4+ coarse speckled	RNP
3 Ps	33, F	OM (SLE) Fever Arthritis Puffy fingers RP	-	P	0.13	-	Empiric	9	218	Hepatitis	Past TB	-	204	No	4+ Coarse speckled	Sm, RNP
4 Hs	23, M	ASSD	1.5	B,Py	0.75	MMF	Synovial Biopsy	-	-	-	CMV proctocolitis, candida, dermatophytosis disseminated, LRI	ILD	-	-	-	-
5 Js	28, F	OM (SLE)	2.0	GI	0.25	HCQ, AZA, ELNT 6 m prior	Ileal biopsy^+^	3	70	Isoniazid psychosis	Pyelonephritis and sepsis	-	57	No	-	-
7 Sk	58, F	DM	12	D*	0.125	-	Pus culture	-	-	-	MAC (M intracellulare pos)	CLD	-	-	-	-
8 Sh	23, F	DM	2	Pl	0.25	-	Empiric	-	-	-		Pregnancy	-	-	-	-
9 As	51, F	DM ILD pneumomediastinum Arthritis Fever	1	P	0.25	MMF	Sputum smear	1	121	Photosensitive rash	LRI, recurrent boils	Confusion between ATT rash Vs. rash of DM	104	Yes	Negative	Negative
10 Hp	42, F	OM (MCTD) Myositis Arthritis RP	5	LN (mediastinal)	0	HCQ, GC	Empiric^~^.	6	72	None	LRI	ILD	66, later developed military TB	Yes	4+coarse speckled	RnP
11 Sc	25, F	OM (MCTD) RP, Sclerodactyly, esophageal dysmotility, ILD, myositis	1.5	P	0.75	MTX, GC 0.5 mg/kg	Sputum smear	9	220	None	-	Vertebral compression fractures	141	No	4+ Rim	RNP
12 Us	40, F	ASSD (anti-Jo-1) Arthritis Myositis	11	R	0.12	-	Renal Biopsy (granulomatous interstitial nephritis), Urine culture positive	1	128	-	Fungal skin infection	-	128	No	Negative	ND
13 An	29 F	DM Rash Myositis	2	LN	0.5	-	Lymph node biopsy	-	-	-	-	-	-	-	-	-
14 Rs	40, F	ASSD Myositis ILD Rash Arthritis MH	0.17	LN (mediastinal)	0	None	Empiric^&^	-	80	Rash	No	ILD	80	No		Ro
15 Ur 3	5, F	ASSD Myositis Arthritis	2	P	0.25	CYC	Empiric	10 m + unknown	84	Pancreatitis and Hepatitis	Herpes Zoster	-	57	No	Negative	Jo-1, SSA
16 Pr	26, F	OM (MCTD) Raynaud’s Arthritis Myositis	0.17	P	0.25	-	Sputum smear	-	-	-	Oesophageal candida, dengue fever	-	-	-	-	-
17 Ar	27, F	DM Rash Myositis	2.5	P	1.5	-	Sputum smear	-	-	-	Pseudomonas sepsis, E coli Urinary tract infection	-	-	-	-	-
18 Dd	31, M	DM Rash Myositis	9	Pl	0.1	MTX	Empiric^#^	-	216	None	Lower respiratory infection at the first admission	Diabetes	108	Yes, biochemical, not clinical	1+ speckled	ND
19 Pi	26, F	PM Myositis	1	P	1	-	Sputum smear	-	-	-	Aspiration pneumonia	ILD	-	--	-	-
20 Ld	55, F	PM Myositis Dysphagia	2	B (Hip)	2.5	MTX	Empiric^@^	12	87	-	-	-	79	No	-	-
22 Ld	50, F	OM (SLE) Fever Arthritis Myositis Seizures	3	D	0.25	-	CSF culture	-	-	-	-	-	-	-	-	-
23 Rs	55, F	PM Myositis	0.67	M,D	1	-	Empiric	-	-	Hepatitis	Cellulitis (finger)	-	-	-	-	-
24 Gp	52, M	DM Rash Myositis	0.75	P	0.5	MTX	Sputum smear	11	69	Transaminitis	Diabetes	None	63	No	4+ homogenous	negative
**Juvenile dermatomyositis**																
21 Mk	18, M	jDM Arthritis Rash Myositis	1	D	0.25	None	Empiric^$^	18 `	58	Transaminitis twice but TB relapsed on treating with modified ATT and later granuloma found in the LN	Staphylococcal sepsis	Seizure-unexplained	TB recurrence at 54 months R resistant, sputum positive, rest awaited	Yes	ANA 2+ fine speckled.	negative
6 Sf	27, M	jDM	-	LN	0.125	-	Empiric	-	-	-	-	-	-	-	-	-

Immunosuppressant (IS) at the time of disease: IS agents in last 6 months.

Flare: within 6 months of last ATT taken.

LN: lymph node; P: pulmonary; B: bone; M: miliary; D: disseminated; R: renal; GI: gastrointestinal; Pl: pleural; Py: pyomyositis; RP: Raynauds’ phenomenon; MTX: Methotrexate; AZA: Azathioprine; MMF: mycophenolate Mofetil; ELNT: Cyclophosphamide as per eurolupus protocol; GC: glucocorticoids; AFB: acid fast bacilli; CSF: cerebrospinal fluid; CXR: chest radiograph; INH: isoniazid; R: rifampicin; Q: fluoroquinolones; E: ethambutol; ILD: interstitial lung disease; MH: mechanic’s hand; ND: not done; ILD: interstitial lung disease; ATT: anti-tubercular therapy; TB: tuberculosis; CLD: chronic liver disease; DM: dermatomyositis; OM: overlap myositis; SLE: systemic lupus erythematosus; CSF: cerebro-spinal fluid; CMV: cytomegalovirus; MAC: mycobacterium avium intracellulare; PM: polymyositis; ASSD: anti-synthetase syndrome; ADRs: adverse drug reaction; GC: glucocorticoid; ANA: anti-nuclear antibodies; ENA: extractable nuclear antigens; MCTD: mixed-connective tissue disorder; ASSD: anti-synthetase syndrome.

“Resistant to INH, R,Q,E and sensitive to aminoglycosides.

* Chest wall abscess and metacarpal

@ Bone biopsy Histopathology and TB PCR negative; MRI: marrow oedema in the left neck of femur with contrast enhancement with minimal reactive hip effusion - probably infective.

$ Multiple cultures and smears negative, including sputum, blood, pericardial and pleural fluid and bone marrow – but axillary lymph node biopsy showed granulomatous inflammation.

& Chest radiograph suggestive of a left hilar shadow and pleural thickening, Computerized tomography of the chest suggested necrotic lymph nodes with pleural thickening.

# Smear and culture of sputum negative but radiology suggestive.

~ IGRA, BACTEC, smear and culture negative, Real time PCR neg; Second time – BAL Smear and culture negative but Mycobacterium tuberculosis complex detected by GeneXpert Assay, Rifampicin sensitive.

` HRZE (29/4/2014 for 2 months) → HR (1 month) → HEL (20 days) → HR (7days) → SEL (3 weeks) → HEL+ Inj Steptomycin (3 months) → HEL (2 months) → HRE (3 years)

+ Gross nodular thickening and stricture, Histopathology granuloma and AFB, TB PCR and culture negative.

### Diagnosis of TB in SLE

The diagnosis of TB was empiric in most cases with adult SLE while in seven paediatric lupus it was based on tissue/smear positivity (**Table 1**). None of them was suspected to be drug resistant.

### The course of TB in IIM

Fourteen were followed up after the diagnosis of TB, of which one was lost after one month of treatment. The median GC dose was 0.25 (0.1–1.8) mg/kg/day at the time of diagnosis of TB. Nine of the 13 followed up (69.2%) had 10 adverse events related to ATT: 5 had drug-induced hepatitis, one had pancreatitis, 2 had a skin rash; one of them was related to para-amino salicylate sodium (PAS) (Two patients had more than one adverse effect).

Median follow-up duration after the diagnosis of TB was 82 (69.3–126.3) months, while median TB free interval was 72.5 (57–107) months. The median duration of ATT was 9 months (5.3–11.2). A resolution of TB was documented in all who followed up. Two patients had a recurrence of TB, at 66 and 54 months respectively, despite taking a complete course of ATT. One of them developed military TB and the other sputum positive resistant TB. There was one death from TB in this cohort. A prolonged course of ATT was required in 25% of patients as compared with 2% in the general population. Confirmed drug resistance was 3%, similar to the general population.

The treatment with ATT resulted in a relapse of myositis in 5 cases, of which flare of muscle disease was seen in 3 patients, while 2 had cutaneous involvement. However, by the last follow up visit, all patients had attained remission.

### The course of Tuberculosis in SLE

Out of 18 cases of TB in patients with SLE, 10 were in remission, five had relapse of SLE during or after completion of ATT and 3 had persistent active disease. Among these two were lost to follow up, 14 were cured of TB and two died. Two out of 290 SLE patients (714 per 100000 patients) suffered mortality, as compared with 32 per 100000 rates in the general population.^2^ SLE patients with tuberculosis suffered 22 times higher mortality than that seen from TB in the general population.

## DISCUSSION

Idiopathic inflammatory myositis is known to be a debilitating disease with a high risk of infections. We have previously described infections as the leading cause of in-hospital mortality in patients with IIM.^1^ Of the various infections, Tuberculosis is one that remains a particular challenge in India. Apart from being a Tuberculosis endemic zone, the emergence of drug-resistant strains has further compounded the picture in this region.

We found that 14% of patients with IIM developed TB, and this risk was 68 times higher than the general population, and 2.24 times higher than SLE patients. Extra-pulmonary, atypical, and disseminated forms of TB were more common. A sizeable number required prolonged courses of ATT, although all improved and prevalence of drug resistance was comparable with the general population.

Although the literature is replete with case reports of TB at odd sites in IIM patients, data on the prevalence of TB in a cohort of IIM in the developing countries is scant.^21^ In a multi-centre study from France published in 2005, of 156 patients with IIM, 18 developed opportunistic infections, of which four had TB.^22^ biochemical findings, and paraclinical features of PM/DM to detect patients at risk of opportunistic infections. Methods The medical records of 156 consecutive PM/DM patients in 3 medical centers were reviewed. Results Eighteen PM/DM patients (11.5% Another one from Finland, on the contrary, reported a high prevalence of 7.4% in 176 patients with IIM.^23^ Of these, 11 (6.25%) were due to M Tuberculosis, while others were atypical Mycobacteria. A Mexican series of 196 myositis found three cases of TB, all extrapulmonary, which occurred in the inactive phase of myositis though within the first five years of illness.^24^ A Taiwanese series of 192 IIM patients reported a prevalence of 3.1% through six cases.^26^ characteristics and predictors of infections in patients with PM and DM.\nMethods. The medical records of 192 PM/DM patients followed up in a tertiary teaching medical centre from 1999 to 2008 were retrospectively reviewed. Seventy-six episodes of major infection, defined as infections requiring >1 week of treatment with anti-microbial agents, occurred in 53 (27.6% On the other hand, a nationwide registry from Taiwan found a prevalence of 1.2% TB in 4958 newly diagnosed DM patients with a hazard ratio of 2.64 after adjusting for age, gender, and the underlying medical disorder.^21^ It is notable that despite sizeable number of overlap IIM, which frequently can be managed with lower doses of glucocorticoids, the prevalence of TB was higher than all other studies reported.^21^

A higher prevalence of TB in IIM and SLE could be related to the use of immunosuppressive drugs which may lead to activation of latent TB infection (LTBI). Wu PH et al. have established that glucocorticoid and azathioprine use as risk factors for TB in a large nationwide study.^21^ Although rituximab use has been considered safer, Gazaix-Fontaine et al. have described TB even after RTX use, suggesting that background disease, as well as TB prevalence in the country, might be contributively.^27^ This merits further exploration in larger multicentre studies. Intravenous Immunoglobulin could be a safe therapy for the management of active diseases in the setting of TB.^28^ Apart from the activation of latent bacilli, de-novo infection could result from an immunosuppressed state. Both SLE and IIM can, at times, have an associated underlying immunodeficiency, more so in children.^29^ Apart from inherited complement defects, most common being C4 deficiency, seen with both SLE and IIM, polymorphisms and copy number variations in C2, C4, and C4A gene have associated with both SLE and polymyositis.^30,31,32^ Of particular note, Mannose Binding Lectin (MBL) deficiency is reasonably common and confers a high risk of Tuberculosis.^33^

The higher prevalence of TB in IIM could also be contributed by a failure to clear pooled secretions. Besides, IIM patients often require higher doses of steroids for more extended periods as compared to SLE. Moreover, LTBI is almost universal in the Indian population; hence screening practice for LTBI is not employed in India.^34^ The only study on the use of prevention in 97 SLE patients in India reported a remarkable decline in Tuberculosis from 11.6% to 2% over a two-year observation period.^35^ Another paper confirmed that in patients who seroconvert for TST while on biologics, Tuberculosis could be prevented with Isoniazid prophylaxis.^36^ Such initiatives can go a long way in reducing both morbidities, and possibly even mortality. Thus, prevention can be considered in the TB endemic setting in patients receiving high dose corticosteroids like patients with IIM, keeping in mind the challenges from polypharmacy and consequent pill burden, drug interactions, and risk of emergence of resistant strains.

Notably, we found various atypical sites of TB in the current study, paralleling patterns seen in other immuno-suppressed states such as HIV. The extrapulmonary TB is common in other rheumatology disease settings such as TNF-inhibitors induced reactivation of TB.^37^ Muscle involvement with TB is infrequent; however, one case of the 24 suggesting a possible preference of the TB bacilli to involve inflamed sites with greater vascularity. Interestingly Nagayama et al. have previously described a case of Tuberculous myo-fasciitis in a patient with DM.^38^ Subsequently, upon review of the eight instances of such Tubercular involvement in literature, three were found to have IIM.^38^ It seems plausible that an inflamed muscle may act as a nidus for infection.

We also noted that a sizeable number of cases developed drug-induced adverse effects. Polypharmacy could complicate the management of TB in IIM. Transaminitis from anti TB treatment can be confused with a relapse of muscle disease, mandating caution while interpreting laboratory tests. With better understanding of the risk, predictors and outcomes of TB in IIM, it may become possible to devise less toxic regimens for management. The strengths of our study include a modest sample size, a comparator group of SLE patients, and follow-up of a subgroup of cases. The limitations are a retrospective nature with the consequent possibility of ascertainment bias as well as under-reporting. There was lack of data on personal history of TB which merits exploration. Further, complete data on immunosuppression received is not available due to retrospective nature of the study. Since the prevalence of TB has gradually changed over the decades with changing treatment practices, disparate time periods of study in the SLE and IIM patients may account for some differences. However, this being the first such study from India assumes relevance as a pilot study. We hope that this exploratory study will pave the way for future prospective studies to understand more about TB in these diseases. The differences in prevalence of TB among various IIM also need to be studied in a prospective study.

Ours being a tertiary care centre receives a referral from more severe illness, and hence prevalence in milder forms of myositis such as amyopathic myositis cannot be ascertained. Besides, the lack of a stringent referral system could have led us to miss cases who reported new symptoms of TB to the general practitioner on follow-up.

## CONCLUSION

Patients with IIM have increased risk of active TB, with common extrapulmonary forms, slower response, and relapses during treatment.
